# Unusual gingival actinomycosis post allogeneic hematopoietic stem-cell transplant: case report

**DOI:** 10.1186/s12903-023-02777-7

**Published:** 2023-02-02

**Authors:** Julia Stephanie Bruno, Wanessa Miranda-Silva, Vitor Heidrich, Marianne de Castro Gonçalves, Yana Novis, Celso Arrais-Rodrigues, Anamaria Aranha Camargo, Eduardo Rodrigues Fregnani

**Affiliations:** 1 Centro de Oncologia Molecular, Hospital Sírio-Libanês, São Paulo, SP Brazil; 2 Departamento de Bioquímica, Instituto de Química, Universidade de São Paulo, São Paulo, SP Brazil; 3 Centro de Oncologia, Hospital Sírio-Libanês, São Paulo, SP Brazil; 4 Hospital Nove de Julho, Rede DASA, São Paulo, SP Brazil; 5 Universidade Federal de São Paulo, São Paulo, SP Brazil

**Keywords:** Oral diseases, Actinomycosis, Oral microbiota, Allogeneic hematopoietic stem-cell transplant

## Abstract

**Background:**

Allogeneic hematopoietic stem cell transplant (allo-HSCT) is used to treat several hematological diseases, but immunosuppression during allo-HSCT facilitates opportunistic microbial growth in tissues, such as actinomycosis. An effective diagnosis of opportunistic diseases is essential for correct management of the disease and preservation of the immunosuppressed patient’s life.

**Case description:**

A 57-year-old female patient was diagnosed with extranodal nasal type NK/T cell lymphoma and underwent curative treatment with allo-HSCT. Twenty-one days after the last clinical follow-up, the patient presented a necrotizing lesion in the papilla region between the first and second molars of the second quadrant. Histopathological analysis showed the presence of a bacterial cluster consistent with Actinomyces infection, and a dense lymphoid infiltrate was also observed. Immunohistochemistry for CD20, CD3, and CD56 was performed to exclude the possibility of the recurrence of extranodal NK/T cell lymphoma. Oral microbiota profiling showed a huge increase in the abundance of Actinomyces bacteria in the subgingival region three weeks prior to appearance of the lesion.

**Conclusions:**

Opportunistic infections with an unusual clinical appearance are confounding factors in therapeutic decision-making. We present for the first time a case of actinomycosis in the gingival papilla region following allo-HSCT. We also highlight how microbiota profiling through next-generation sequencing could be used to anticipate bacterial infection diagnosis.

## Background

Allogeneic hematopoietic stem-cell transplant (allo-HSCT) recipients undergo a conditioning regimen to induce immunosuppression and prevent graft rejection. However, a less competent immune system puts patients at risk of opportunistic microbial infections. In the oral cavity, patients can present herpes simplex infection, candidiasis, and bacterial infections after allo-HSCT [1, 2].

*Actinomyces* is a prevalent bacterial genus in the oral cavity, mainly found in the periodontal region [3]. As *Actinomyces sp*. do not possess genes coding for decomposition enzymes, such as hyaluronidases, no tissue degradation is inflicted on the host under normal conditions. However, epithelial barrier injury caused by dental procedures or mucosal ulceration makes the oral cavity susceptible to opportunistic infections caused by *Actinomyces sp.* (i.e., actinomycosis). In line with this, the oral cavity accounts for 50–60% of all actinomycosis cases, afflicting the jaw region primarily. It is characterized by the presence of abscess and mandibular osteomyelitis [4]. Other susceptible sites include the pelvic-abdominal region and lungs, comprising 20% and 10% of actinomycosis cases, respectively [4–6]. Few cases report actinomycosis in the allo-HSCT setting [6, 7]. To the best of our knowledge, the case described here is the first with necrotizing aspects in the papillary region of the gingiva.

A rapid diagnosis of opportunistic infections during cancer treatment is essential for better management of the infection and prevention of treatment discontinuation. In addition to reporting a case with a unique appearance in the soft tissue of the oral cavity, we demonstrate that oral bacteria tracking through 16S rRNA sequencing can prompt an early diagnosis, anticipating clinical tissue disorder.

## Case presentation

**Fig. 1:**
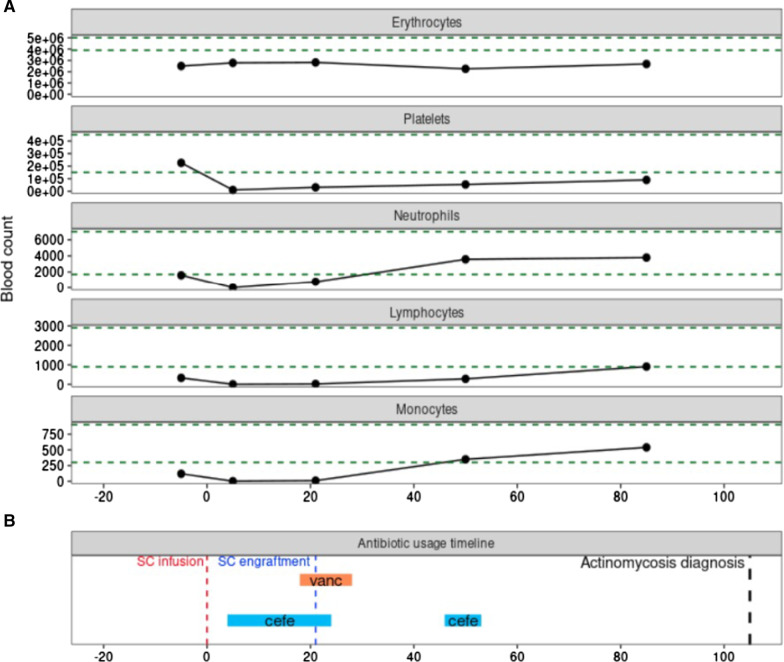
Blood cell counts and antibiotics administered during allogeneic hematopoietic stem-cell transplant (allo-HSCT). **A** Blood cell counts during allo-HSCT. Blood cell count data was collected at the following allo-HSCT timepoints: D-5 (preconditioning), D5 (aplasia), D21 (engraftment), D50 (~ 4 weeks after engraftment), and D85 (~ 9 weeks after engraftment). Dashed green horizontal lines represent normal reference values. **B** Antibiotic usage timeline during allo-HSCT. Antibiotics used between stem-cell (SC) infusion and actinomycosis diagnosis: cefepime (cefe) and vancomycin (vanc)

A female patient aged 57 years was diagnosed with extranodal nasal type NK/T cell lymphoma, cancer staging (EC) IVA, with cutaneous and central nervous system infiltration. The patient underwent allo-HSCT. The graft source was the first-degree sister's bone marrow and the conditioning regimen was performed with Fludarabine-Melphalan and Total Body Irradiation at 400 cGy. Graft-versus-host disease protocol included cyclophosphamide + mycophenolate mofetil (D90) + cyclosporin A. Patient's blood counts (erythrocytes, platelets, lymphocytes, neutrophils, monocytes) at critical allo-HSCT timepoints and antibiotics usage are represented in Fig. 1.

Dental evaluation before hematological treatment did not identify lesions in the oral mucosa. Dental elements were also in good condition with adapted amalgam restorations. The patient reported discomfort in the oral mucosa during allo-HSCT and developed xerostomia grade II (NCI CTCAE v 3.0) and mucositis grade II (WHO grade) in the retropharyngeal region. In addition to primary oral care, the patient received photobiomodulation with low-level laser equipment (Laser XT Therapy, DMC, São Carlos, Brazil) at a wavelength of 660 nm (spot-size = 0.028 cm2; 100 mW of power) to aid in the healing of oral mucositis. Healing occurred within 12 days after the onset of ulceration.

### Oroscopy

**Fig. 2:**
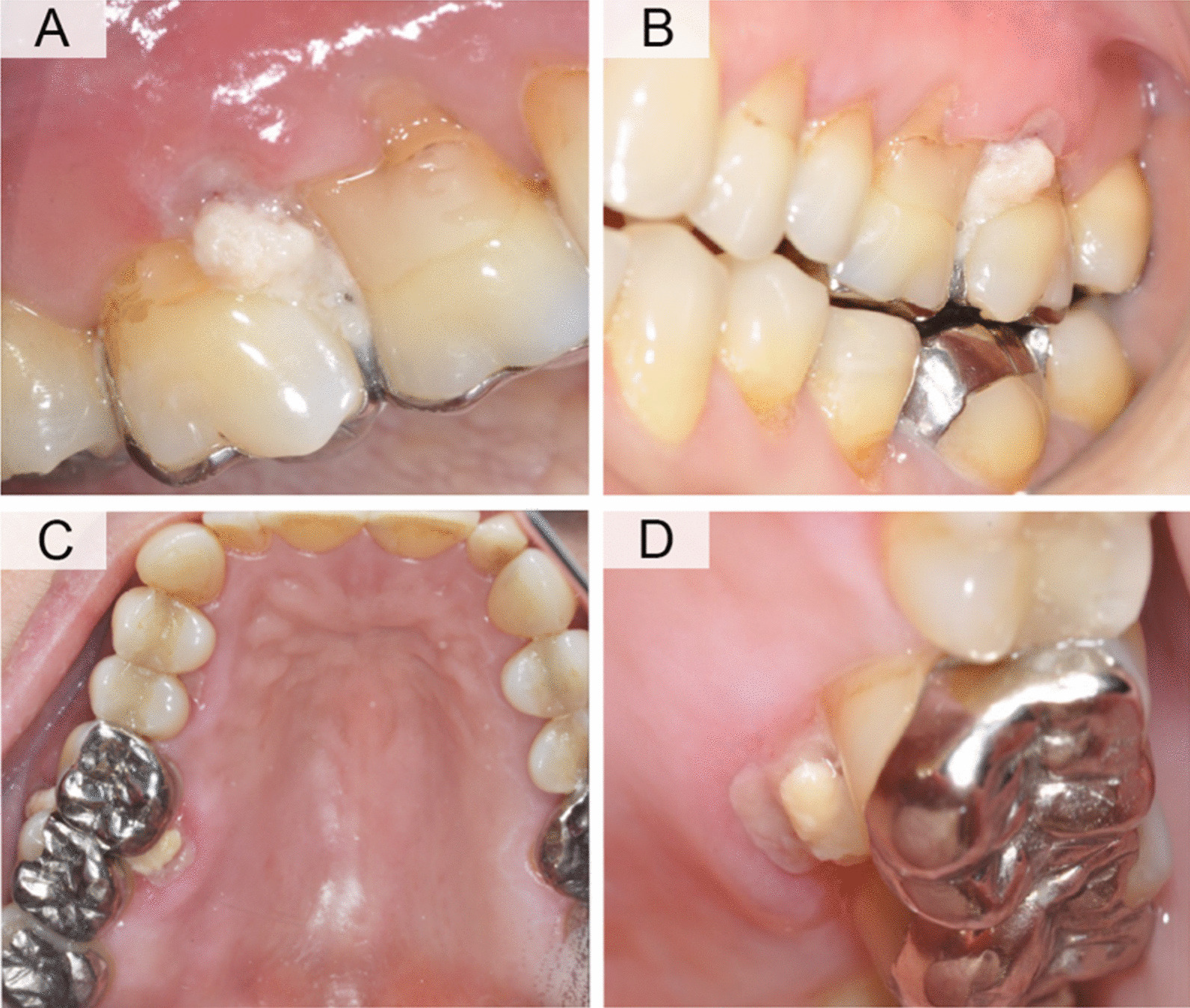
Different perspectives of oral lesion clinical aspects. **A** mesial, **B** vestibulo-mesial, **C** palatal and **D** mesio-palatal

Twenty-one days after the last clinical follow-up, the patient presented a necrotizing lesion in the papilla region between the first and second molars of the second quadrant and no involvement of the rest of the hard palate or alveolar ridge. A biopsy was performed to confirm the diagnosis of an opportunistic infection in the region of the erythematous border and necrotizing area (Fig. 2).

### Histopathological analysis

**Fig. 3:**
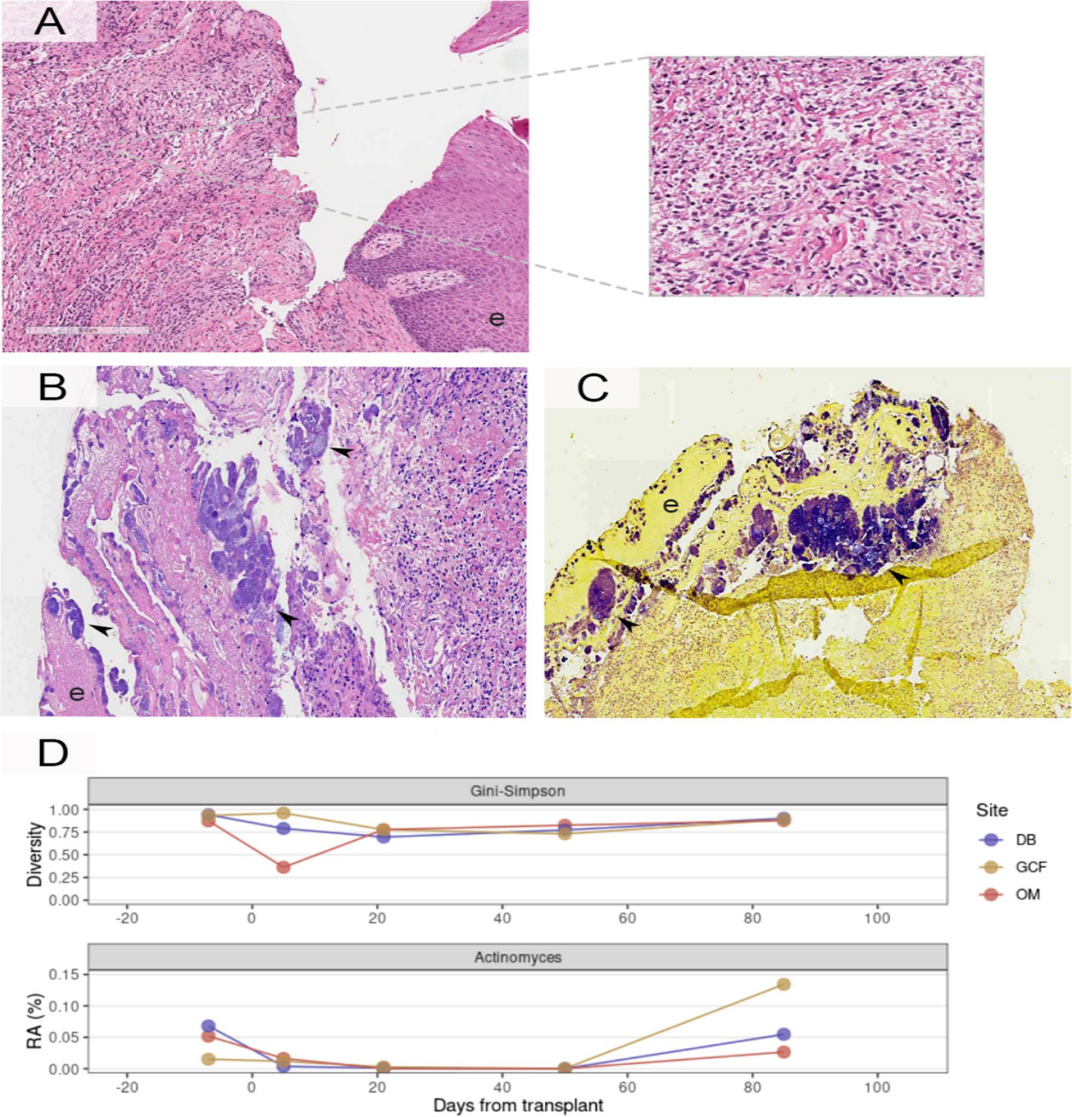
Pathological and molecular studies. **A** Histological study between the erythematous border and deepithelialization demonstrating inflammatory reaction with lymphocytes and collagen fibres (HE, left × 100 and right × 300); **B** Histological study in the central portion of the lesion with necrotizing inflammation and accumulation of colonies of gram-positive bacteria (arrow); **C** Gram stain histology demonstrating positivities for gram-positive bacteria with coccoid-shaped colonies (GS, × 100); **D** Oral microbiota profiling. Upper graph: alpha diversity (Gini-Simpson index) throughout transplantation and follow-up. Bottom graph: with relative abundance (RA) of the genus *Actinomyces*. *DB* dental biofilm, *GCF* gingival crevicular fluid, *OM* oral mucosa (*HE* hematoxylin and eosin, *GS* gram-staining, *NGS* next-generation sequencing)

**Fig. 4:**
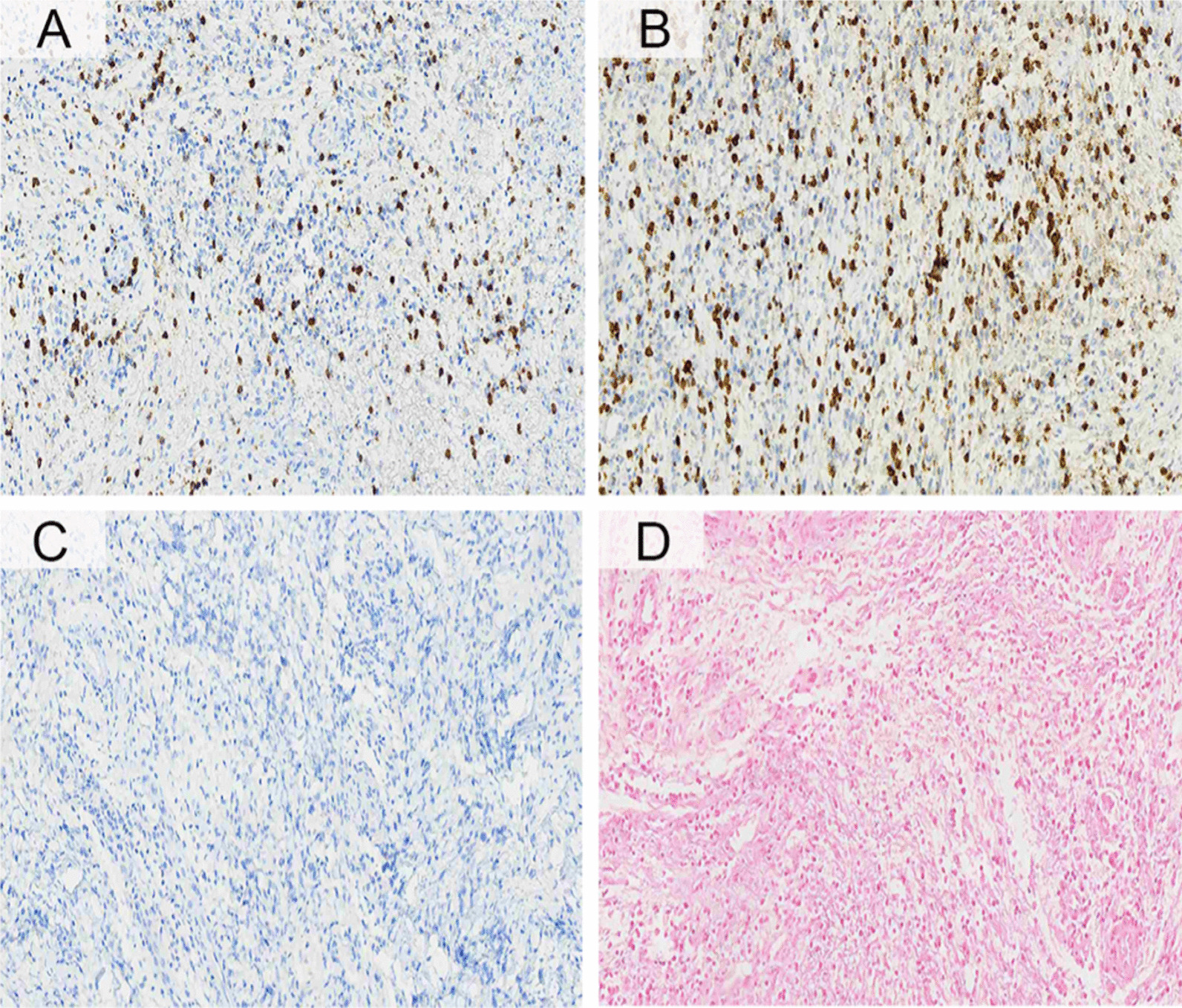
Study of extranodal NK/T-cell lymphoma markers. Immunohistochemistry method for **A**–**C** and, in situ hybridization for viral status. **A** CD20, **B** CD3, **C** CD56 and **D** Epstein–Barr virus (EBV)

Histopathological analysis showed ulcerated mucosa with dense lymphoid infiltrate with intermediate size lymphocytes, fibrosis, some plasma cells, and rare eosinophils (Fig. 3A). The presence of a bacterial cluster consistent with *Actinomyces* infection was also observed (Fig. 3B, C). Immunohistochemistry for CD20, CD3, and CD56 was performed to exclude the possibility that the lesion in the gingiva was a recurrence of extranodal NK/T cell lymphoma. There was a mixed infiltrate of mature B and T lymphocytes without atypia. As evaluated by in situ hybridization, the tissue was negative for Epstein-Barr virus (Fig. 4). After the histopathological analysis results, the patient was treated with amoxicillin (875 mg)-potassium clavulanate (125 mg), every 12 h for 4 weeks. There is no photographic record of the healed oral mucosa. The patient died due to aggressive lymphoma recurrence 93 days after the Actinomycosis diagnosis.

### Oral microbiota analysis

This patient was enrolled in a research protocol for the longitudinal profiling of the oral microbiota during allo-HSCT (Research Ethics Committee-Hospital Sírio-Libanês: #HSL 2016-08). Oral mucosa (OM), dental biofilm (DB), and gingival crevicular fluid (GCF) samples were collected at: D-5 (preconditioning), D5 (aplasia), D21 (engraftment), D50 (~ 4 weeks after engraftment), and D85 (~ 9 weeks after engraftment). Samples were processed and sequenced for microbiota profiling as described previously [8, 9]. Oral microbiota profiling showed drastic diversity changes at all oral sites during allo-HSCT with higher decrease in OM, but similar diversity levels were maintained across all oral sites during follow-up (Fig. 3D). As expected, *Actinomyces* was detected at high relative abundance in all oral sites before allo-HSCT, but abundance decreased during transplant. In the last sample analysed, there was an increase in *Actinomyces* abundance at all oral sites, particularly in GCF. *Actinomyces* relative abundance in the gingival crevicular fluid was 890% higher in D85 than in D-5, while other oral sites showed slight decreases compared to pre-allo-HSCT (− 20% in DB and − 49% in OM). The increase in *Actinomyces* abundance in GCF preceded the appearance of oral lesions by 20 days. Noteworthy, a single sequence variant of *Actinomyces* (100% identity with *Actinomyces oris*, *Actinomyces naeslundii,* and *Actinomyces viscosus*) was mainly responsible for this abundance increase, suggesting the involvement of a single or a few closely related opportunistic species.

## Discussion

*Actinomyces* bacteria are gram-positive, filamentous bacilli. They compose the commensal microbiota of the oral cavity, genitourinary tract, and gastrointestinal tract in humans. In opportunistic situations, *Actinomyces* causes tissue destruction in the lung, bone, genitourinary tract, digestive tract, central nervous system, skin, and mucosa [4, 10]. Cervicofacial actinomycosis, represents 60% of cases [4]. Immunosuppression, poor oral hygiene, age, male sex, and malnutrition are risk factors for the progression of opportunistic lesions [4]. Strategies for maintaining oral microbial eubiosis should be further studied to contain the growth of periodontopathogens [11].

A correct actinomycosis diagnosis is crucial to contain the progression of abscesses and osteomyelitis [4] and can be life-saving for immunosuppressed patients. However, actinomycosis is often hard to diagnose, especially when it mimics other diseases, such as cancer, tuberculosis, and nocardiosis. The diagnosis by bacterial cultivation can be challenging. Since *Actinomyces* bacteria are part of the commensal polymicrobial community of the oral cavity and periodontal regions, contamination during swab collection can result in false-positive results. Therefore, sample collection for detecting oral actinomycosis must be performed carefully, preferably by sampling the inflammatory exudate or the tissue biopsy. Also, following sample collection*,* due to *Actinomyces* microaerophilic or strict anaerobic characteristic, samples need to be quickly processed in a controlled laboratory environment to prevent false-negative results [4]. False-negatives are even more common in the oncological setting because immunosuppressed patients receive continuous prophylactic antibiotics, which, in addition to a long *Actinomyces* incubation time (average of two weeks), can mislead cultivation results. Histological studies may help the diagnosis in some cases by the detection of sulfur granules, which are responsible for keeping the bacterial colony protected from phagocytosis [4, 10].

Opportunistic infections with an unusual clinical appearance are confounding factors in therapeutic decision-making. Drug therapy in immunosuppressed patients, as well as rapid diagnosis, can be decisive to maintain the patient's systemic stability and oncological follow-up [4, 10]. We here detail a case of an unusual necrosing manifestation of actinomycosis in the gingival papilla. We showcase how microbiota profiling through next-generation sequencing can be an essential tool to anticipate tissue necrosis progression by tracking changes in oral microbes abundance.

## Data Availability

The raw sequencing data analysed during the current study are available at https://github.com/vitorheidrich/oral-microbiota-actinomycosis.

## Abbreviations

allo-HSCT: Allogeneic hematopoietic stem-cell transplant
OM: Oral mucosa
DB: Dental biofilm
GCF: Gingival crevicular fluid
